# WHO cone bioassay boards with or without holes: relevance for bioassay outcomes in long-lasting insecticidal net studies

**DOI:** 10.1186/s12936-022-04412-2

**Published:** 2022-12-19

**Authors:** Melanie Koinari, Nakei Bubun, Brogan Amos, Kiari Kiari, David Lahu, Stephan Karl

**Affiliations:** 1 Australian Institute of Tropical Health and Medicine, James Cook University, Smithfield, QLD Australia; 2 Vector-Borne Diseases Unit, Papua New Guinea Institute of Medical Research, Madang, Madang Province Papua New Guinea

**Keywords:** Cone bioassay, Long-lasting insecticidal nets, LLIN, Pyrethroid, Bioassay board, Holes, Mosquito, *Anopheles*, *Aedes*

## Abstract

**Background:**

The World Health Organization (WHO) cone bioassay is a key method used to evaluate the bioefficacy of long-lasting insecticidal nets (LLINs) used for malaria control. These tests also play an important role in LLIN product prequalification and longitudinal monitoring. Standardization of these assays is therefore important. While many parameters for WHO cone bioassays are defined in the respective WHO guidelines, others are not. One of these undefined parameters is the exact configuration of the bioassay boards. In cone bioassays, LLIN samples are pinned onto a bioassay board for testing. Anecdotal evidence suggests that bioassay boards with holes behind the LLIN samples lead to greater exposure to insecticide, as the mosquitoes are ‘forced to stand on the net material’. This may increase the key assay outcomes of 60 min knockdown (KD60) and 24 h mortality (M24). The present study tested this hypothesis in two facilities using two fully susceptible mosquito colonies.

**Methods:**

WHO cone bioassays were performed using bioassay boards with holes and boards without holes in parallel, following WHO guidelines. Five brands of LLINs with four new and unwashed whole net samples per brand were used (total of n = 20 whole nets). Five pieces per whole net sample were prepared in duplicate resulting in a total of n = 100 pairs.

Knock-down (KD) was recorded in 10 min intervals within the first hour after exposure and mortality was recorded at 24 h. Assays with *Anopheles farauti* were done at the Papua New Guinea Institute of Medical Research (PNGIMR) and assays with *Aedes aegypti* were done at James Cook University, Australia.

**Results:**

Results varied not only with bioassay board configuration but also with mosquito colony. In particular, with *An. farauti,* a significantly higher M24 was observed when boards with holes were used, while this was not observed with *Ae. aegypti.* WHO cone bioassay results were systematically biased between the two facilities such that the use of *An. farauti* at PNGIMR predicted higher KD60 and M24.

**Conclusion:**

The present study highlights the need for further harmonization of WHO cone bioassay methodology. Parameters such as bioassay board configuration and mosquito species systematically affect the observations, which impedes generalizability of WHO cone bioassay outcomes.

**Supplementary Information:**

The online version contains supplementary material available at 10.1186/s12936-022-04412-2.

## Background

Long-lasting insecticidal nets (LLIN) are a key vector control tool for the prevention of malaria in endemic countries. They are estimated to have saved millions of lives [1, 2]. LLIN procurement volumes have continuously increased since widespread mass distributions began in the early 2000s. Annual LLIN deliveries are now approaching 300 million pieces, while the cost per net has more than halved in the last 10 years [3]. The increasing demand for cheap LLINs, the rapid spread of pyrethroid resistance and competition for LLIN market share between manufacturers has diversified the product palette in recent years [4–6]. Alongside much needed progress (e.g., with new and innovative active ingredient (AI) formulations), this increased market complexity also led to challenges for LLIN product quality assurance, and occurrence of substandard products [4, 7–12]. Recipient countries and donors increasingly recognize that LLINs are not just ‘mosquito nets’ but complex public health commodities that require stringent quality assurance and performance monitoring [4, 12, 13].

WHO published guidelines for testing LLINs, initially in 2005 [14]. In 2013, these guidelines were updated and augmented with additional guidance for monitoring the durability of LLINs under ‘operational conditions’ [15]. The declared aims of the WHO guidelines are (1) to provide specific standardized procedures for testing of LLINs and (2) to harmonize standard testing procedures [15].

Tests for LLIN bioefficacy under laboratory conditions, i.e., the ability of LLIN products to kill mosquitoes in cone bioassays, are an integral part of the WHO testing guidelines and play a crucial role in the LLIN product prequalification process and, increasingly, in post-market surveillance [4, 7, 12, 16–18].

Given that the market fate of LLIN products depends on their performance in these bioassays, which are also part of long-term durability and wash–resistance evaluations, it is important that they are standardized [4, 13].

Since total AI content is not correlated with bioefficacy, bioassays are crucial to evaluate product performance. Bioassay tests usually involve the exposure of living mosquitoes to LLIN product material, and thus, even in their simplest form, these tests require a complex experimental setup including the maintenance of a mosquito colony [19, 20].

The simplest bioassay recommended to evaluate LLIN products for bioefficacy is the WHO cone bioassay [15, 21]. WHO guidelines outline in detail how cone bioassays should be performed, providing important parameters such as sample size, number of mosquitoes to be exposed, number of replicates to be performed, exposure time, and acceptable temperature and humidity ranges. However, other key parameters that may also affect the key cone bioassay endpoints of 60 min knockdown (KD60) and 24 h mortality (M24) are either undefined or only partially defined by WHO. An obvious example for this is that the mosquito species to be used in WHO cone bioassays is currently not further specified. The WHO guidelines require ‘susceptible’ (female) *Anopheles* mosquitoes [15, 21]. Yet, it is evident that ‘susceptibility’ does not mean the same (or even similar) response of different mosquito species to standardized exposure to insecticides. That is why guidance for mosquito susceptibility testing is species and genus specific [22]. In other words, using different mosquito species (and potentially even different mosquito strains) in bioassays is synonymous with systematic bias. This situation can be likened to the known species-dependent mosquito responses observed in WHO tube bioassays used for insecticide resistance monitoring and the recognized need for species-specific, WHO-recommended, discriminatory insecticide concentrations to be used in these assays [22, 23]. It is thus surprising that species-specific guidance for each prequalified LLIN product is lacking, as the declared priorities of harmonization and standardization of bioassays would clearly require this level of rigor.

**Fig. 1:**
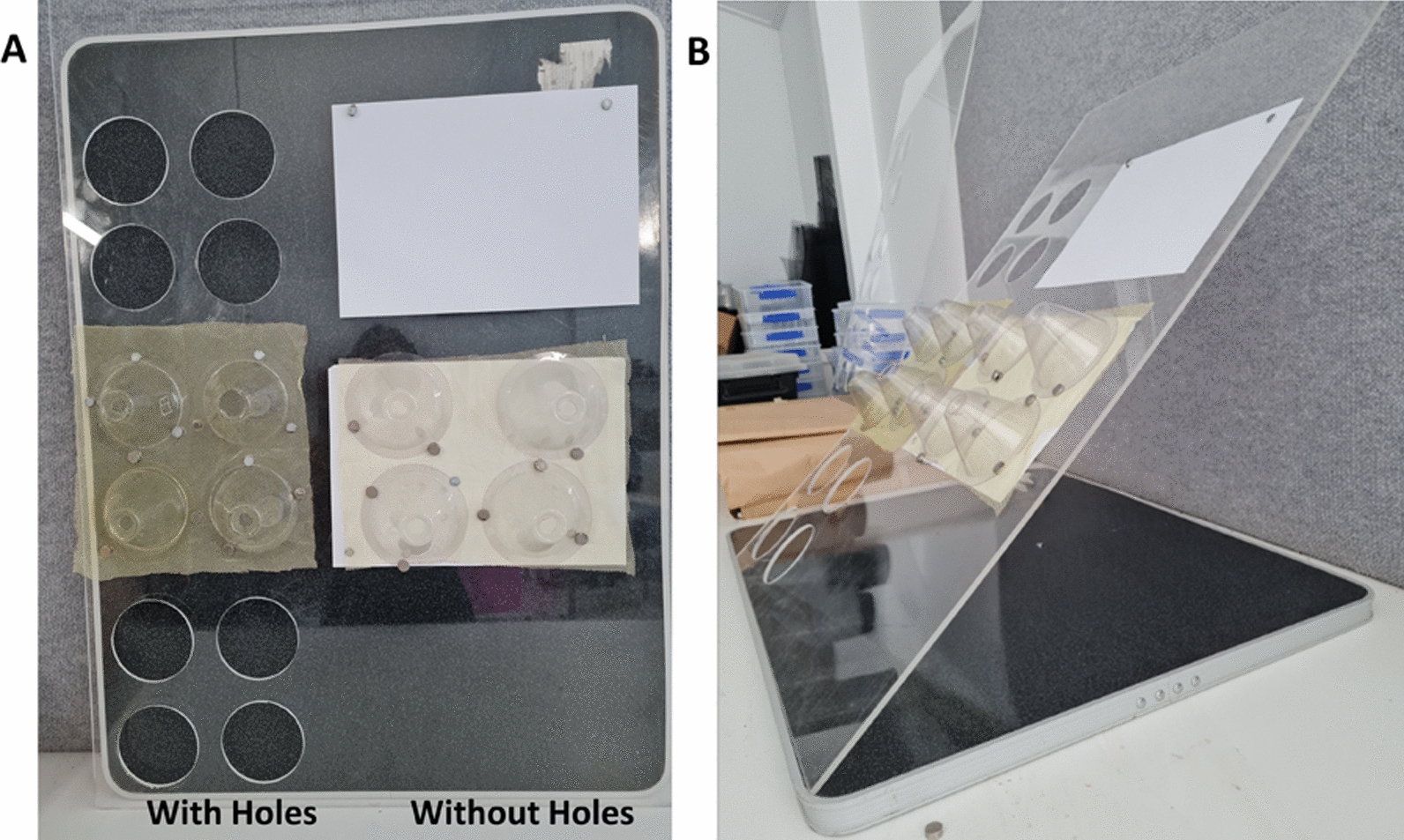
WHO cone bioassays conducted using bioassay board configurations ‘with holes’ and ‘without holes’ sections. Panel **A**: Four WHO cones were placed on a net, which is placed over the ‘holes’ or over an A4 sheet of paper (‘no holes’)**.** Four cones on each net sample (replicates 1–4). Panel **B**: The board assembly was placed against the wall at an angle of 45°

Besides the use of different mosquito species, other important bioassay parameters are also left open for interpretation or are not standardized even if the relevant guidelines are explicit. As a result, testing laboratories may be inclined to ‘optimize’ these undefined or less stringently enforced bioassay parameters to achieve the highest bioefficacy outcomes (i.e., high KD60 and M24). This stands in contrast to the overarching aim of ‘harmonization’ and ‘standardization’ as it may further increase systematic bias between testing facilities and lead to situations where LLIN products may routinely ‘pass’ testing criteria in some settings but not in others. One such undefined (or partially defined) set of parameters is the configuration of the bioassay board to which the LLIN samples are pinned for the duration of the test. While this may seem trivial, even here, multiple parameters may crucially influence bioassay results. For example, while WHO guidelines state that these boards should be placed at a 45 degree angle, Owusu et al., 2016, compared different angles for positioning of the cone bioassay board set up and found that mosquitoes (pyrethroid susceptible *Anopheles gambiae* Kisumu-1 and pyrethroid resistant *Anopheles stephensi* STI) spent more time on the nets at a 60 degree angle [24]. As a result, some studies are now reconfiguring this experimental detail in order to maximize the measured bioefficacy indicators of M24 and KD60 [20, 25]. Another such modification is the use of bioassay boards with circular holes i.e., the material behind the tested sample being removed, again with the intention to ‘maximize exposure’ and thus increase bioefficacy endpoints (Fig. 1). While WHO guidelines do not specify whether this should be done or not and the authors are not aware of studies having systematically quantified the resulting effect, several laboratories have conducted WHO cone bioassays on boards with holes cut [20, 24] but others did not [12, 20].

Therefore, the present study, conducted in 2021 and 2022, investigated whether circular holes in the bioassay boards intended to ‘force the mosquitoes to stand on the net surface’ (as shown in Fig. 1) lead to systematic bias in the KD60 and M24 key bioassay endpoints.

## Methods

### Study locations

The study was conducted at two facilities (i) the Vector-borne Diseases Unit of the PNG Institute of Medical Research (PNGIMR), using fully pyrethroid susceptible *Anopheles farauti* colony mosquitoes and (ii) the Mosquito Research Facility at James Cook University (JCU), using fully susceptible *Aedes aegypti* mosquitoes.

### Source of LLINs

Five groups of LLINs were included in the present study as shown in Table 1. All were unused and unwashed, and obtained in original packaging. LLINs with manufacturing year, 2019 and 2020, were sampled from deliveries for mass distribution immediately upon arrival in Papua New Guinea (PNG). These were requested to be tested for bioefficacy by the National Malaria Control Programme and Rotarians Against Malaria PNG who implement LLIN mass distributions in the country. Unused PermaNet^®^ 2.0 nets manufactured in 2012 were obtained from the Madang Provincial Health Authority. Two groups of PermaNet^®^ 2.0 samples were included as they exhibit substantially different bioefficacy as shown in previous studies [12, 26]. More details about the individual LLIN samples are provided in Additional File 1.

**Table 1:** LLIN samples included in the present study

N	Net Brand	Material	Active Ingredient	Denier	Manufacture Year
4	PermaNet^®^ 2.0	Polyester	1.8 g/kg Deltamethrin	75	2012
4	PermaNet^®^ 2.0	Polyester	1.8 g/kg Deltamethrin	75	2019
4	Interceptor^®^	Polyester	5 g/kg alpha-Cypermethrin	100	2020
4	Yorkool^®^ LN	Polyester	1.8 g/kg Deltamethrin	75	2019
4	SafeNet^®^	Polyester	5 g/kg alpha-Cypermethrin	100	2019 & 2020

This study used n = 4 whole net LLIN samples from n = 5 manufacturers (i.e., a total of n = 20 whole net samples). Each whole net sample was prepared for WHO cone bioassays as per WHO guidelines [13, 14], cutting n = 5 pieces (one from each side and one from the roof) in duplicate. This resulted in a total number of n = 100 duplicate (paired) LLIN pieces for testing.

From each of the 100 pairs, one piece was retained at PNGIMR and one piece was sent to JCU. Net pieces were wrapped individually in aluminum foil, placed inside zip lock plastic bags and stored at 4 °C until processed.

### WHO cone bioassay

Cone bioassays with LLINs were conducted according to WHO guidelines [15] using fully susceptible *An. farauti *sensu stricto (*s.s.)* mosquitoes (Rabaul strain) [12] at PNGIMR and fully susceptible *Ae. aegypti* (W_melb_ strain) mosquitoes at JCU. The experimental set up of the WHO cone bioassays is shown in Fig. 1. Two identical bioassay boards (dimensions 900 × 600 x 5 mm) were prepared from clear acrylic. The boards were divided into two sections and three sets of four holes (same diameter as the bioassay cones’ diameter) were cut in one of the sections.

Cone bioassays were performed on a board ‘without holes’ (as per WHO guidelines) and ‘with holes’ in parallel (Fig. 1). A net piece was placed directly over the ‘holes’ and four bioassay cones were attached to it using small magnets. On board sections ‘without holes’, an A4 piece of paper was placed between the board and the net piece and, likewise, four cones were attached to it using magnets. The boards were set up against a wall at a 45° degree angle [24] before mosquitoes were introduced into the bioassay cones. Cone bioassays for of each LLIN were performed on both the ‘with hole’ and ‘without hole’ configurations of the bioassay boards. Boards were washed with unscented soap and water, rinsed and dried in the sun in between assays.

Using an aspirator, 5 insecticide susceptible, non-blood-fed, 2–5 day old female *An. farauti* (PNGIMR) or *Ae. aegypti* (JCU) mosquitoes were introduced into a cone and a cotton ball was used to plug the hole. Mosquitoes were exposed to the net pieces for 3 min (timed individually for each cone), after which they were gently transferred from the cones to a holding cup screened with untreated netting and provided access to 10% sugar solution via a soaked piece of cotton wool placed on top of the netting. Knockdown was recorded at 10, 20, 30, 40, 50 and 60 min after exposure. Mortality was recorded at 24 h after exposure. Mosquitoes were scored as ‘alive’ if they were able to both stand upright and fly in a coordinated manner; knocked-down if it could not stand (e.g. had one or two legs), could not fly in a coordinated manner (e.g., taking off briefly but falling down immediately) and as dead if it was immobile, and showed no signs of life. An untreated polyester net was used as negative control. Cone bioassays at PNGIMR were performed under laboratory conditions with 28.5 ± 2.7 °C and 65 ± 9% relative humidity, whereas at JCU temperature was 26.0 ± 2.5 °C with a relative humidity of 75 ± 11%.

### Data analysis

Data were analysed using Microsoft Excel 2016 (Microsoft Inc.) and GraphPad Prism 9.3.1 (GraphPad Software). The main endpoints of WHO cone bioassays are KD60 and M24, however, knockdown kinetics were also compared as an additional endpoint. WHO standard bioefficacy criteria for cone bioassays for pyrethroid-only LLIN are ≥ 95% KD60 and/or ≥ 80% M24.

**Fig. 2:**
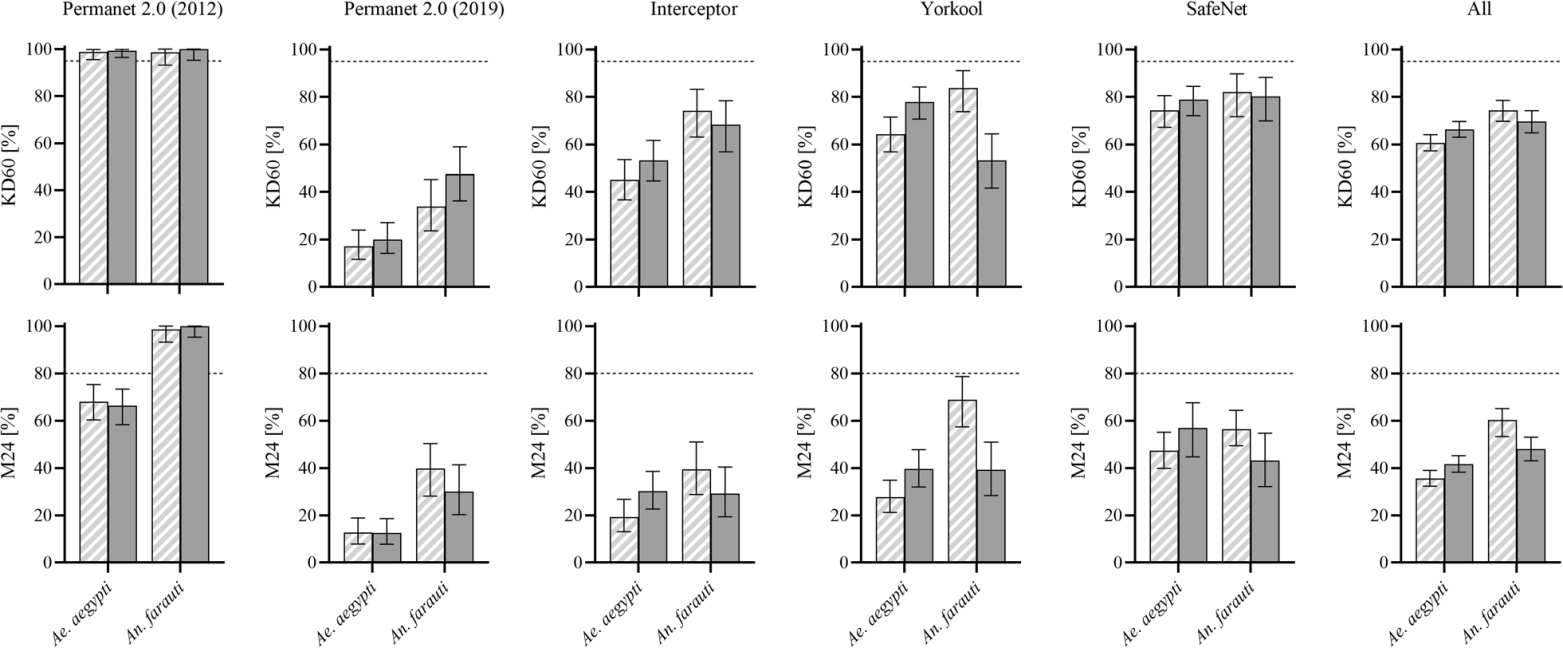
Percent knockdown and mortality of *Aedes aegypti* and *Anopheles farauti* after exposure to different LLIN brands. Panels show 60 min knock down (KD60, top row) and 24 h mortality (M24, bottom row) for each LLIN type tested. Error bars are 95% confidence intervals of proportions

**Fig. 3:**
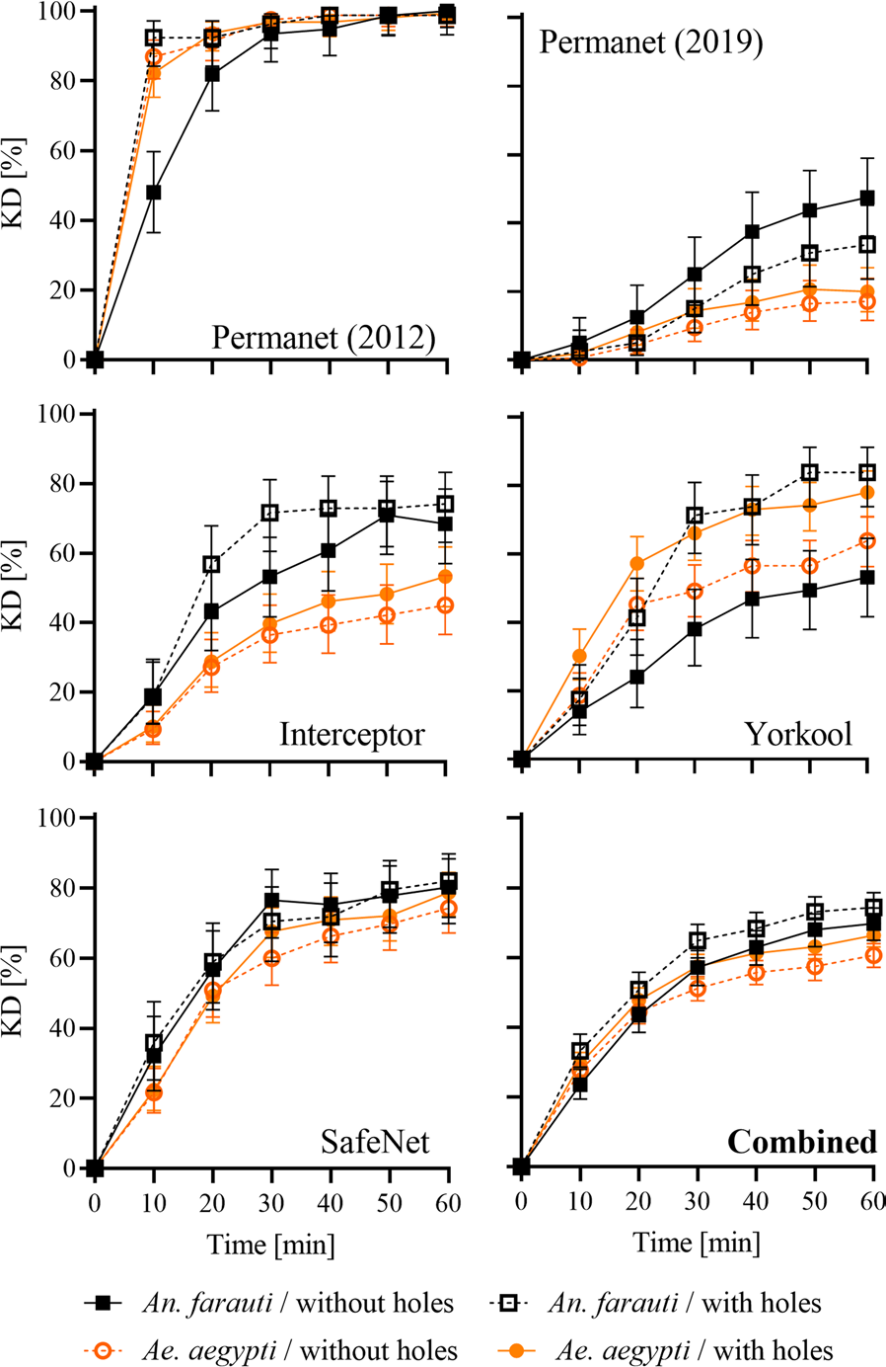
Knockdown kinetics after exposure measured in 10 min intervals until 60 min post exposure. Panels show knockdown kinetics for each LLIN product tested and also for all products combined, and for the different bioassay conditions (mosquito strains and bioassay board configuration). Error bars represent mean and 95% confidence intervals of proportions

To summarize proportion data (Figs. 2 and 3), mean proportions and 95% confidence intervals of proportions were used. To compare population proportions between boards with holes and without holes, or between facilities (mosquito species), two-sample Z tests of proportions were used. For correlation data, non-parametric correlation statistics (Spearman) correlation was used.

## Results

### Effect of holes in bioassay boards on knockdown and mortality rates

Results obtained with WHO cone bioassays using bioassay board configurations with holes and without holes and using either *An. farauti* or *Ae. aegypti* mosquitoes are summarized for each LLIN type and overall in Table 2. The table shows the percent KD60 and percent M24 for all mosquitoes tested with each net type (i.e., the tests for individual nets and net panels combined).

**Table 2:** Summary of test results using bioassay boards with and without holes

KD60	*Ae. aegypti*		*An. farauti*	
	*Holes*	*No holes*	*Holes*	*No holes*
PermaNet 2.0 (2012)	98.8% (95.6–99.9)	99.4% (96.5–99.9)	98.7% (93.2–100.0)	100% (95.3–100)
PermaNet 2.0 (2019)	17.1% (11.6–23.9)	20% (14.1–27.0)	33.8% (23.6–45.2)	47.5% (36.2–59.0)
Interceptor	45.0% (36.6–53.6)	53.2% (44.6–61.7)	74.1% (63.1–83.2)	68.4% (56.9–78.4)
Yorkool	64.4% (56.9–71.5)	77.8% (70.7–84.2)**	83.8% (73.8–91.1)	53.2% (41.6–64.5)***
SafeNet	74.3% (67.2–80.6)	78.8% (72.1–84.5)	82.1% (71.7–89.8)	80.3% (69.9–88.3)
Average	60.7% ( 57.3–64.1)	66.4% (63.0–69.7)*	74.4% (69.8–78.6)	69.7% (64.9–74.2)
M24	*Ae. Aegypti*		*An. Farauti*	
	*Holes*	*No holes*	*holes*	*No holes*
PermaNet 2.0 (2012)	68.1% (60.3–75.3)	66.2% (58.3–73.4)	98.7% (93.2–100.0)	100% (95.3–100)
PermaNet 2.0 (2019)	12.7% (7.9–18.9)	12.5% (7.8–18.6)	39.8% (28.1–50.3)	30% (20.3–41.3)
Interceptor	19.3% (13.1–26.8)	30.2% (22.7–38.6)*	39.5% (28.8–51.0)	29.1% (19.4–40.4)
Yorkool	27.7% (21.2–34.9)	39.6% (32.0–47.7)*	68.8% (57.4–78.7)	39.2% (28.4–50.9)**
SafeNet	47.4% (39.8–55.1)	57.0% (49.4–64.4)	56.4% (44.7–67.6)	43.1% (32.2–54.7)
Average	35.6% (32.3–39.0)	41.7% (38.2–45.2)*	60.3% (53.3–65.1)	48.0% (43.0–53.0)**

Results are presented as proportions (in %) and their 95% confidence intervals (in parentheses). Resulting KD60 and M24 for the different bioassay board configurations were compared using Z-tests of proportions

^*^0.01 < p < 0.05; **0.001 < p < 0.01;***p < 0.001

Interestingly, there were slightly opposite tendencies in the effect that the holes in the bioassay boards had on WHO cone bioassay endpoints, depending on the mosquito species used. For *Ae. aegypti*, there was a small (around 5%), and mildly statistically significant (0.01 < p < 0.05, Z-test to compare proportions) decrease in observed KD60 and M24 when boards with holes were used. For *An. farauti,* there was a moderate increase in M24 of around 12%; p = 0.002) when bioassay boards with holes were used.

Mosquito species had a pronounced small-to-medium systematic impact on the WHO cone bioassay outcomes, and using pyrethroid susceptible *Ae. aegypti* resulted in systematically lower KD60 (63.5% vs 72.0%, Cohen’s h = 0.18, p < 0.0001) and M24 (38.6% vs 54.2%, Cohen’s h = 0.31, p < 0.0001) as compared to using pyrethroid susceptible *An. farauti*. Interestingly, this effect was most prominent and apparent across all products when bioassay boards with holes were used. In this group (bioassay boards with holes), M24 for *An. farauti* was much higher (35.6% vs 60.3%, Cohen’s h = 0.5, P < 0.0001), and KD60 was also significantly increased (p = P < 0.0001) as compared to *Ae. aegypti*. The results are graphically presented in Fig. 2.

Figure 3 shows the knock down kinetics in the 60 min after exposure as measured in 10 min intervals for the two bioassays board configurations (with and without holes) and the two mosquito species. Some LLIN types seemed to invoke larger differences between board configurations and/or species than others did. However, with the exception of Yorkool LN (*An. farauti*, without holes) and PermaNet 2.0 2012 (*An. farauti*, without holes), the general pattern of *Ae. aegypti* resulting in consistently lower KD values is evident.

**Fig. 4:**
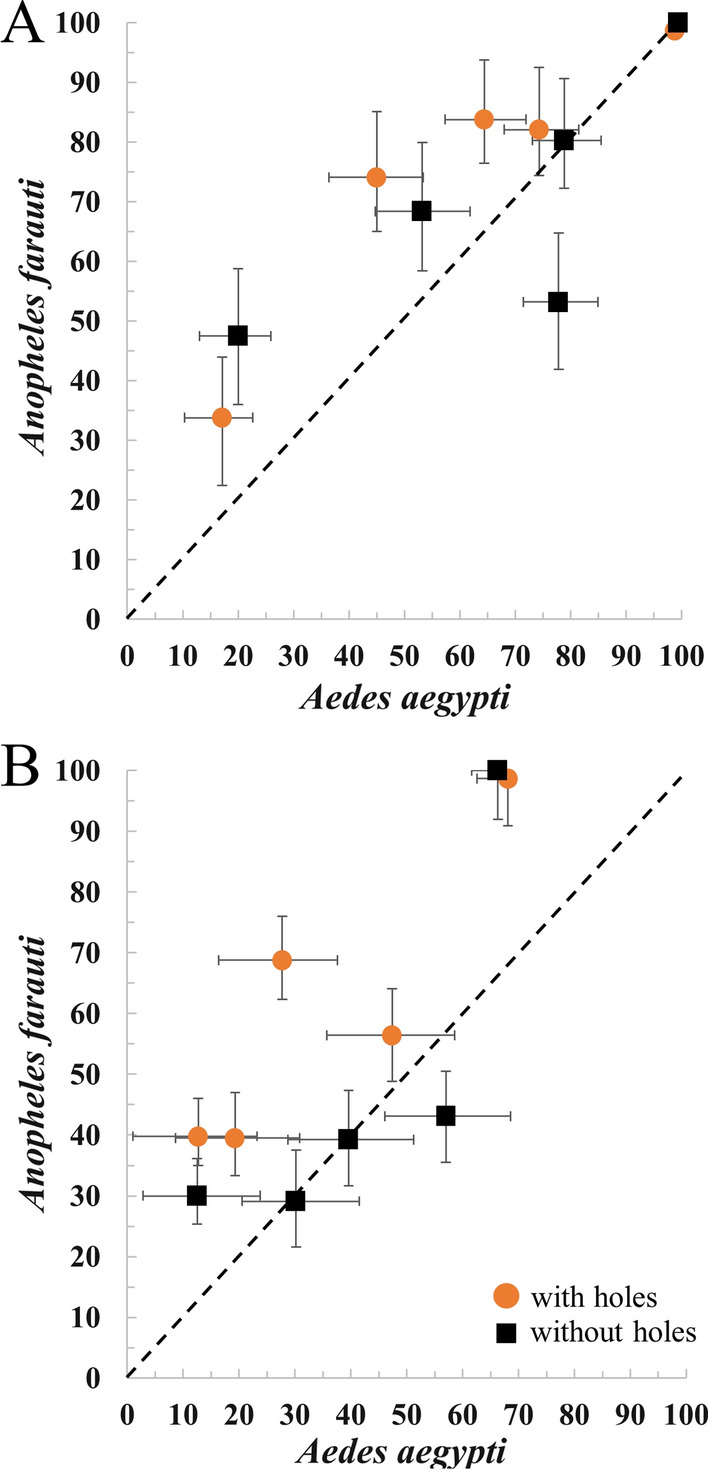
Correlation of cone-bioassay data for *Aedes agypti and Anopheles farauti*. Panel **A**, 60 min knockdown (KD60) and Panel **B**, 24 h mortality (M24) endpoints compared between *Ae. agypti* and *An. farauti.* Bioassay board configuration is indicated by the shape and colour of the symbols (black square: without holes; orange circle: with holes). The diagonal dashed line is a line of identity and meant to be a guide to the eye. Experiments with *Ae. agypti* were conducted at JCU and experiments with *An. farauti* were conducted at PNGIMR using the same LLIN samples but on different net cuttings

Figure 4 shows a correlation analysis for KD60 and M24 endpoints between mosquito species. The figure again illustrates that for *An. farauti*, bioassays resulted in systematically higher KD60 and M24 outcomes and that this effect was more pronounced when bioassay boards with holes were used, whereas, for *Ae. aegypti* overall KD60 and M24 were lower and the differences caused by the bioassay configuration were smaller (i.e., the points for bioassays conducted on boards with no holes are closer to the line of identity).

## Discussion

Overall, these results demonstrate that pyrethroid susceptible *An. farauti* are more sensitive to bioassay board configuration changes as compared to susceptible *Ae. aegypti,* and that the combination of bioassay board configuration and mosquito species can result in quite substantial systematic differences in the key WHO cone bioassay outcomes of KD60 and M24. For example, in this study, overall M24 in the bioassay board configuration group of ‘with holes’, was 36% for *Ae aegypti* but 60% for *An. farauti* (p < 0.0001).

WHO cone bioassays are used to evaluate and compare LLIN products, with the intention to ‘*harmonize testing procedures in order to generate data for registration and labelling’* [15]. The present results demonstrate that this desired standardization is currently not being achieved because important parameters are not defined in the current testing guidelines [15]. Bioassay outcomes can currently only be interpreted taking into consideration the mosquito species that was used and other important parameters such as the configuration of the bioassay boards. As a consequence, it is questionable if WHO cone bioassays in their current form should be used to evaluate products against fixed, universal bioefficacy performance thresholds such as 95% KD60 and 80% M24 across different settings.

This does not mean that cone bioassays conducted in different settings are not equally meaningful and reproducible, but as testing laboratories use different mosquito strains and different bioassay configurations, often with the intention to maximize exposure (and thus, bioefficacy endpoints) the outcomes obtained in different settings are bound to be systematically biased.

While variation of factors that can easily be standardized such as bioassay board configuration could be avoided, other factors are much harder to standardize, given the complexity and number of parameters involved. In addition, some settings may simply not be able to use specific mosquito species due to regulatory restrictions. As such, it seems very unlikely that full standardization of WHO cone bioassays is achievable or desirable across settings. A better option may be to enable harmonization between settings, by conducting dedicated multi-centre studies to establish baseline parameters defining the systematic bias between settings. The authors have recently conducted such a study between PNGIMR and the Ifakara Health Institute (IHI), Tanzania [20]. This study showed that the systematic bias in observed 24 h mortality values in both facilities was 17 percentage points (95% CI −27 to + 63 percentage points), i.e., 24 h mortality observed at IHI was on average 17 percentage points lower as compared to PNGIMR, when two *Anopheles* species colonies (*An. gambiae* and *An. farauti*) were used [20].

Other WHO documents e.g., those used for insecticide resistance monitoring provide mosquito species-specific guidance [22]. However, since other factors such as temperature [27] have been reported to potentially influence bioassay outcomes, it is questionable if mosquito species-specific bioefficacy thresholds are the solution to the harmonization/standardization problem.

A limitation of the present study is that other laboratory-specific differences that cannot easily be controlled for between settings may have influenced the results, such as rearing conditions, and maybe temperature and humidity. However, this also adds value to the current study as it more closely resembles ‘real-world’ scenarios experienced in multi-centre LLIN evaluation studies.

This study demonstrates that products should be evaluated against a representative range of mosquito species (including non-anopheline species) and it seems crucial to establish reference bioefficacy parameters for products in order to more generally interpret bioassay results obtained in specific settings. Reference samples and facility-specific reference bioefficacy data can help to detect bioefficacy shifts in products that are indicative of real performance changes due to e.g., changes in product specifications, as it has been demonstrated in LLINs delivered to Papua New Guinea [12].

While it was not part of this study to assess individual products against WHO performance thresholds, it is noteworthy, that only one of the 5 tested products (PermaNet^®^ 2.0 from 2012) resulted in either > 80% average M24 or > 95% average KD with the fully susceptible *Anopheles* strain used in the present study. In contrast, all of these products achieved 100% or near 100% KD60 and M24 when they were evaluated by WHO Pesticide Evaluation Scheme [16–18, 28, 29]. The products used in the present study were all coated polyester nets and thus belong to the same class of products. Even though, WHO guidelines are for anopheline mosquitoes, many studies have also used non-anopheline mosquitoes for LLIN evaluation [30–40]. *Aedes aegypti* colonies are easier and cheaper to maintain, and the extended and more formal use of *Ae. aegypti* (and other model organisms) for insecticide treated net (ITN) testing may be warranted [34]. It is, therefore, important to include non-anopheline mosquitoes in comparative analyses such as the present study. Further studies to assess mosquito genus, species and strain specific responses in cone bioassays are needed. Other factors that may influence the outcome of WHO cone bioassays, such as rearing conditions and environmental parameters should be more extensively and formally investigated. In addition, there is an urgent need to develop complementary physico-chemical analysis methods to reliably quantify and characterize the bioavailable proportion of insecticide on LLIN surfaces to complement cone bioassays.

The fact that systematic bias is strikingly evident, even for the simplest of bioassays available to test LLIN products makes it very likely that more complex assays, such as the tunnel test and experimental huts, with many more undefined parameters (including exposure time, bait animal, mosquito age, tunnel and hut configurations), are even more sensitive to these issues and therefore less likely to predict similar results across different settings. ‘Next generation’ LLIN products that employ AIs with different modes of action (such as Interceptor® G2 [36]) or varying AI composition and concentrations on different net sections (such as PermaNet® 3.0 [41]), require further specific refinement of the testing guidelines that incorporate these complexities in order to enable a meaningful evaluation.

## Conclusions

WHO cone bioassay outcomes are systematically biased depending on the pyrethroid susceptible mosquito species employed and bioassay board configuration. Harmonization of testing procedures across different settings requires quantification of the systematic bias between different settings and setting-specific reference bioefficacy data are needed. Reference samples for each product should be retained at prequalification.

## Supplementary Information

**Additional file 1. Table S1.** Cone bioassay testing of long-lasting insecticidal nets at the two different laboratories.

## Data Availability

The data set for this study is available on request from the corresponding author.

## Abbreviations

AI: Active ingredient
IMR: Institute of Medical Research
KD60: Knockdown after 60-min
ITN: Insecticide treated nets
JCU: James Cook University
LLINs: Long-lasting insecticidal nets
M24: Mortality at 24-h
PNG: Papua New Guinea
WHO: World Health Organization
